# Change in knee cartilage components in stroke patients with genu recurvatum analysed by zero TE MR imaging

**DOI:** 10.1038/s41598-022-07817-w

**Published:** 2022-03-08

**Authors:** Wenshan Li, Youwei Li, Qiang Gao, Jingxin Liu, Qiping Wen, Shiqi Jia, Fen Tang, Linhong Mo, Yuanfang Zhang, Mingchun Zhai, Yukun Chen, Yue Guo, Weijun Gong

**Affiliations:** 1 Beijing Rehabilitation Medicine Academy, Capital Medical University, Beijing, 100144 China; 2 Department of Radiology, Beijing Rehabilitation Hospital, Capital Medical University, Beijing, 100144 China; 3 Scientific Research Department, Beijing Rehabilitation Hospital, Capital Medical University, Beijing, 100144 China; 4 Department of Neurological Rehabilitation, Beijing Rehabilitation Hospital, Capital Medical University, Beijing, 100144 China

**Keywords:** Biological techniques, Diseases, Medical research

## Abstract

Genu recurvatum in stroke patients with hemiplegia causes readily cumulative damage and degenerative changes in the knee cartilage. It is important to detect early cartilage lesions for appropriate treatment and rehabilitation. The purpose of this cross-sectional study was to provide a theoretical basis for the early rehabilitation of hemiplegia patients. We used a zero TE double-echo imaging sequence to analyse the water content in knee joint cartilage at 12 different sites of 39 stroke patients with genu recurvatum and 9 healthy volunteers using a metric similar to the porosity index. When comparing the hemiplegic limb vs. the nonhemiplegic limb in patients, the ratios of the deep/shallow free water content of the femur cartilages at the anterior horn (1.16 vs. 1.06) and posterior horn (1.13 vs. 1.25) of the lateral meniscus were significantly different. Genu recurvatum in stroke patients with hemiplegia can cause changes in the moisture content of knee cartilage, and the changes in knee cartilage are more obvious as the genu recurvatum increases. The "healthy limb" can no longer be considered truly healthy and should be considered simultaneously with the affected limb in the development of a rehabilitation treatment plan.

## Introduction

Approximately 70% of stroke patients have hemiplegia, of whom 40–60% have knee hyperextension^1^. Long-term hyperextension of the knee changes the mechanical trend of the lower limbs, and the knee joint load-bearing reaction worsens such that the stability of the standing phase decreases and the body leans forwards. These changes induce an uneven distribution of the internal stress in the knee joint, cause knee pain and cartilage injury when walking in this way for a long time, and lead to cumulative damage and degenerative changes in knee cartilage^2^. Other tissues around the knee joint, such as the meniscus, also undergo pathological changes^3^. Therefore, there is no doubt that hemiplegic patients with knee reflexes will develop early knee injury. At present, there is no effective drug to treat cartilage injury and repair cartilage defects^4^. If the lesion is detected by imaging before irreversible damage occurs to the articular cartilage and appropriate treatment is adopted, normal knee function can be maintained, and disability can be avoided. Therefore, it is important to detect early lesions of the cartilage.

Cartilage is mainly composed of chondrocytes and extracellular matrix. The extracellular matrix is mainly composed of water, collagen fibres and proteoglycans, and water accounts for 80% of the wet weight of cartilage^5^. In osteoarthropathy, the initial histological and biochemical changes in cartilage are collagen fibre breakdown, decreased proteoglycan content, and increased water dispersion^6^. Therefore, early cartilage lesions manifest as an increased moisture percentage, which is associated with the loss or damage of matrix components^7^. The detection of moisture changes in cartilage would be helpful for the early detection of cartilage lesions.

Although the gold standard for imaging diagnosis in osteoarthropathy is X-ray examination with the Kellgren–Lawrence grading system, it cannot be used to directly assess cartilage. Recently, MRI has become a powerful tool for the examination of early changes in tissue components in vivo and has been used to evaluate osteoarthropathy^8,9^. Traditional MRI uses T2WI and T2 mapping to image cartilage^10,11^, but these MRI sequences can only image long TE tissues and the superficial and intermediate layers of articular cartilage, while the deep and calcified layers cannot be imaged^12^, so they cannot reveal the pathological changes of the deep and calcified layers, especially the early changes^13^. Ultrashort TE sequences overcome this problem by imaging both long and short TE tissues^14^. Recently, many scholars have used these sequences to study articular cartilage, especially UTE T2* mapping and T1 rho mapping, which can quantitatively analyse early changes in endochondral injury^15–18^. However, these techniques are difficult to apply in clinical practice because of their complexity, long scanning time and the influence of the magic angle effect. Some scholars used the porosity index of the double-echo UTE sequence to evaluate the porosity of the bone cortex, calculated as the ratio of the image intensities of the second echo (indicating signals from pore water) and the first echo (indicating signals from all water), and found it to be highly consistent with the results measured by micro-CT^19,20^. This method is relatively simple and has potential for clinical use.

The contrast and signal-to-noise ratio of short TE tissue imaging with a zero TE sequence are basically the same as those of the ultrashort TE sequence^21^. The difference between them is that the readout gradient of the ultrashort TE sequence starts at the beginning of data acquisition, while that of the zero TE sequence starts before the radiofrequency pulse.

No literature has reported changes in early knee cartilage in stroke patients with hemiplegia and knee retraction on MRI. In this study, a zero TE double-echo imaging sequence was used to analyse the water content in the knee joint cartilage of stroke patients with genu recurvatum using a metric similar to the porosity index, and the study focused on three questions: (1) is there any difference between the knee joint cartilage of the hemiplegia side and the nonhemiplegia side in stroke patients? (2) Are the changes in knee cartilage related to the degree of genu recurvatum? (3) Is there any difference between the knee cartilage of stroke patients and normal people? This study aims to provide a theoretical basis for the early rehabilitation of hemiplegia patients.

## Results

### Patients

A total of 151 stroke patients with hemiplegia received rehabilitation treatment in the Neurological Rehabilitation Center of the Beijing Rehabilitation Hospital from January 2019 to October 2020. A total of 39 patients with complete data met the study criteria, including 30 males and 9 females, ranging in age from 32 to 60 years, with an average age of 49 years, and a body mass index (BMI) ranging from 21 to 31 kg/m^2^. Among the 39 patients, three had no available gait data, 9 did not have genu recurvatum (< 5°), 15 had mild genu recurvatum (5°–10°) and 12 had severe genu recurvatum (> 10°). There were 9 healthy volunteers, 5 males and 4 females, ranging in age from 35 to 50 years old, with an average age of 42.5 years and a BMI between 20 and 26 kg/m^2^. There were no significant differences in age, BMI or sex distribution between stroke patients and healthy volunteers (P > 0.05). The morphology and signal of the knee cartilage and meniscus were normal on conventional MRI. The paralyzed limbs, time of illness and walking duration of the patients were recorded (Table 1). Regardless of the handedness and the side of the paralyzed limb, all patients received loading practice and gait training for both limbs, since they were both affected.

**Table 1:** Stroke patient characteristics.

Paralyzed limb	Number of patients	Sex		Time of illness (months)	Walking time (months)	Handedness	
		Male	Female			Left	Right
Left limb	18	12	6	6–30	3–15	0	18
Right limb	21	18	3	6–28	3–18	9	15

One patient with a right paralyzed limb was ambidextrous.

### Volunteer versus patient

Among the patients with right limb hemiplegia, the affected limbs were compared with the corresponding limb of healthy volunteers. The deep free-water content of the tibia cartilage at the anterior horn and posterior horn of the lateral meniscus and the femur cartilage at the anterior horn of the medial meniscus of the affected limb were decreased. The content of free water in the deep layer of the tibial cartilage at the posterior horn of the medial meniscus was decreased, and the content of binding water in the shallow layer was also decreased. However, the free water content in the shallow layer and the binding water in the deep layer of the femur cartilage at the posterior horn of the lateral meniscus of the affected limb was also decreased. The content of free water in the load-bearing cartilage of the medial femoral condyle of the affected limb was relatively increased. When the relatively healthy limb of the patients with right limb hemiplegia was compared with the corresponding limb of healthy volunteers, the deep binding water of the tibia cartilage at the posterior horn of the medial meniscus of the relatively healthy limb was reduced. At the femur cartilage at the posterior horn of the medial meniscus, the free water content in the shallow layer of cartilage was decreased, while the free water content in the deep layer increased and the combined water was also decreased. The free water content in the shallow layer of the femur cartilage at the posterior horn of the lateral meniscus was decreased. The deep free water content in the load-bearing cartilage of the medial femoral condyle of healthy limbs was relatively high.

When comparing the affected limb of the patients with left limb hemiplegia with the corresponding limb of healthy volunteers, we found that the deep binding water of the tibia cartilages at the anterior horn and posterior horn of the medial meniscus and the femur cartilage at the posterior horn of the medial meniscus of the affected limb were decreased. The free water content in the shallow layer of the femur cartilage at the posterior horn of the lateral meniscus, the tibia cartilage at the posterior horn of the medial meniscus and the load-bearing area of the lateral condyle of the tibia was decreased. When the relatively healthy limbs of the patients with left limb hemiplegia were compared to the corresponding limbs of healthy volunteers, the deep free water content of the femur cartilage at the anterior horn of the medial meniscus, the tibia cartilage at the anterior horn and posterior horn of the lateral meniscus, and the tibia cartilage at the posterior horn of the lateral meniscus of the relatively healthy limb were lower, and the free water content of the shallow layer of the tibia cartilage at the anterior horn of the lateral meniscus was also lower. The free water content in the shallow and deep layers of cartilage in the lateral condyle of the tibia of the relatively healthy limbs was decreased.

**Figure 1:**
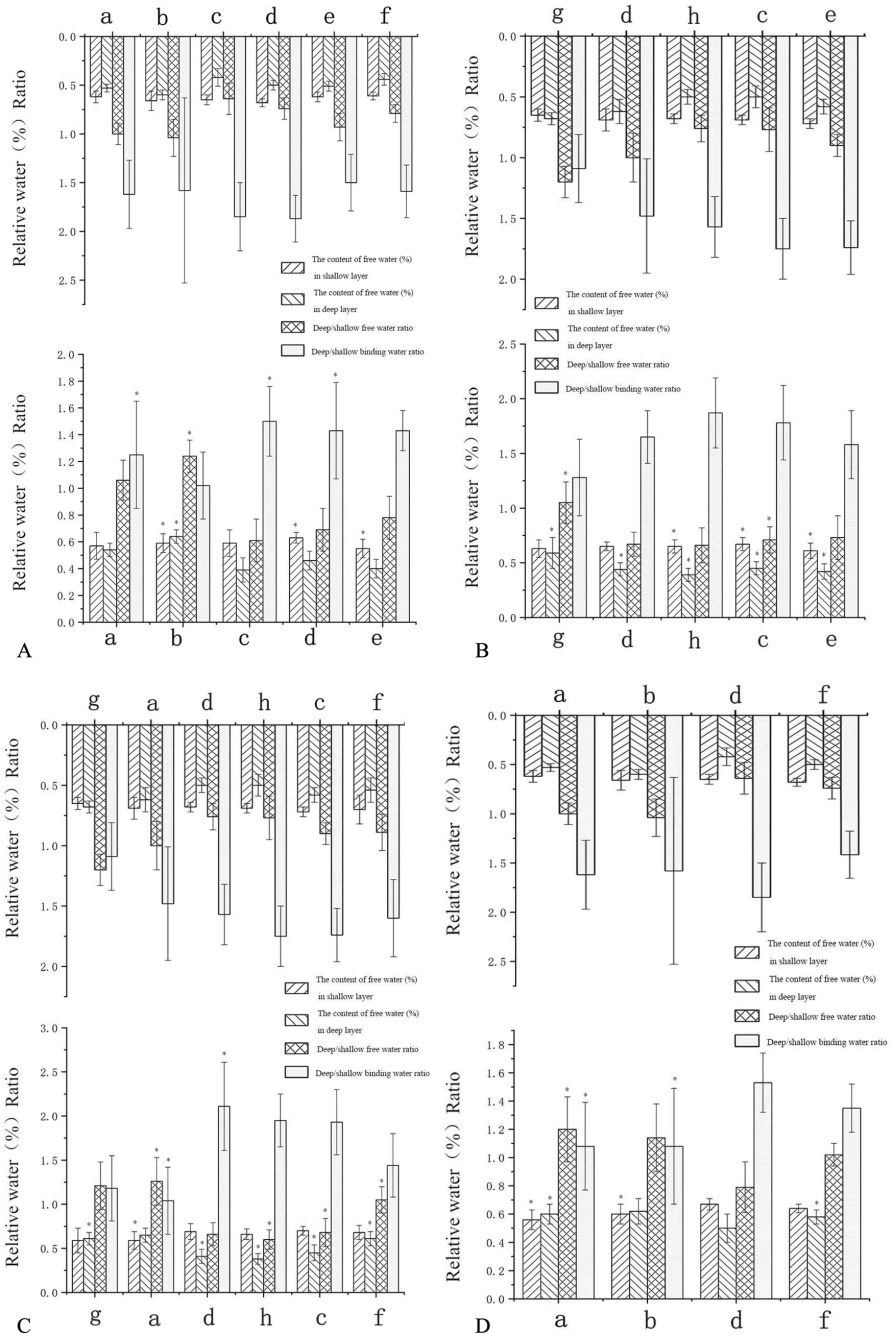
Histograms display statistically significant knee cartilages between hemiplegic patients and healthy volunteers, in which one or more of the four indices had p < 0.05 (*). In the patients with left limb hemiplegia, the hemiplegic limbs (**A**) and the healthy limbs (**B**) were compared with the corresponding limbs of the healthy volunteers. The hemiplegic limbs (**C**) and the healthy limbs (**D**) in patients with right limb hemiplegia were compared with the corresponding limbs of the healthy volunteers. a, The femur cartilage at the posterior horn of the medial meniscus; b, the femur cartilage at the posterior horn of the lateral meniscus; c, the tibia cartilage at the anterior horn of the medial meniscus; d, the tibia cartilage at the posterior horn of the medial meniscus; e, the thickest bearing cartilage of the lateral tibial condyle; f, the thickest bearing cartilage of the medial femoral condyle; g, the femur cartilage at the anterior horn of the medial meniscus; h, the tibia cartilage at the anterior horn of the lateral meniscus.

The changes in the above indicators were statistically significant at *P* < 0.05 (Fig. 1).

### Hemiplegic limb vs. nonhemiplegic limb

There were significant differences in the ratio of the deep/shallow free water content of the femur cartilage at the anterior horn and posterior horn of the lateral meniscus and the deep/shallow binding water content of the tibia cartilage at the anterior horn and posterior horn of the medial meniscus (*P* < 0.05) (Table 2). The deep free-water content of the tibia cartilage at the anterior horn of the medial meniscus and that of the cartilage at the posterior horn were higher in the nonhemiplegic limbs, while the combined water content of cartilage was higher in the hemiplegic limbs.

**Table 2:** Comparison of affected and healthy limbs among hemiplegic patients.

Cartilage	Index	Healthy limb (39)	Affected limb (39)	t	P
Femur cartilage at the anterior horn of the lateral meniscus	Content of shallow free water (%)	0.64 ± 0.07	0.65 ± 0.08	− 0.106	0.915
	Content of deep free water (%)	0.59 ± 0.06	0.61 ± 0.08	− 0.985	0.328
	Deep/shallow free water ratio	1.06 ± 0.16	1.16 ± 0.19	− 2.548	0.013
	Deep/shallow binding water ratio	1.36 ± 0.38	1.69 ± 2.39	− 0.856	0.394
Femur cartilage at the posterior horn of the lateral meniscus	Content of shallow free water (%)	0.59 ± 0.09	0.61 ± 0.08	− 1.198	0.235
	Content of deep free water (%)	0.64 ± 0.07	0.61 ± 0.07	1.816	0.073
	Deep/shallow free water ratio	1.25 ± 0.21	1.13 ± 0.22	2.494	0.015
	Deep/shallow binding water ratio	1.03 ± 0.32	1.20 ± 0.51	− 1.749	0.085
Tibia cartilage at the anterior horn of the medial meniscus	Content of shallow free water (%)	0.62 ± 0.10	0.69 ± 0.08	− 3.052	0.003
	Content of deep free water (%)	0.41 ± 0.10	0.45 ± 0.08	− 1.921	0.058
	Deep/shallow free water ratio	0.63 ± 0.15	0.67 ± 0.14	− 1.243	0.218
	Deep/shallow binding water ratio	1.61 ± 0.30	1.96 ± 1.03	− 2.030	0.048
Tibia cartilage at the posterior horn of the medial meniscus	Content of shallow free water (%)	0.66 ± 0.07	0.66 ± 0.04	0.034	0.973
	Content of deep free water (%)	0.43 ± 0.08	0.47 ± 0.08	− 2.139	0.036
	Deep/shallow free water ratio	0.67 ± 0.14	0.73 ± 0.16	− 1.759	0.083
	Deep/shallow binding water ratio	1.80 ± 0.56	1.58 ± 0.23	2.4227	0.030

### Comparison between patients with and without genu recurvatum

In the affected limbs, the content of free water in the shallow layer of the tibia cartilage at the anterior horn of the lateral meniscus and the femur cartilages at the anterior horn and posterior horn of the lateral meniscus were higher in the patients with genu recurvatum than in those with no genu recurvatum. In patients with genu recurvatum, the deep binding water of the femur cartilage at the anterior and posterior horns of the lateral meniscus was higher than in the patients with no genu recurvatum, while the deep binding water of the tibia cartilage at the posterior horn of the medial meniscus was lower than in the patients with no genu recurvatum. In addition, the deep free water of the femur cartilage at the posterior horn of the medial meniscus was lower than that in patients with no genu recurvatum, while the load-bearing cartilage at the lateral condyle of the tibia was higher than that in patients with no genu recurvatum.

**Figure 2:**
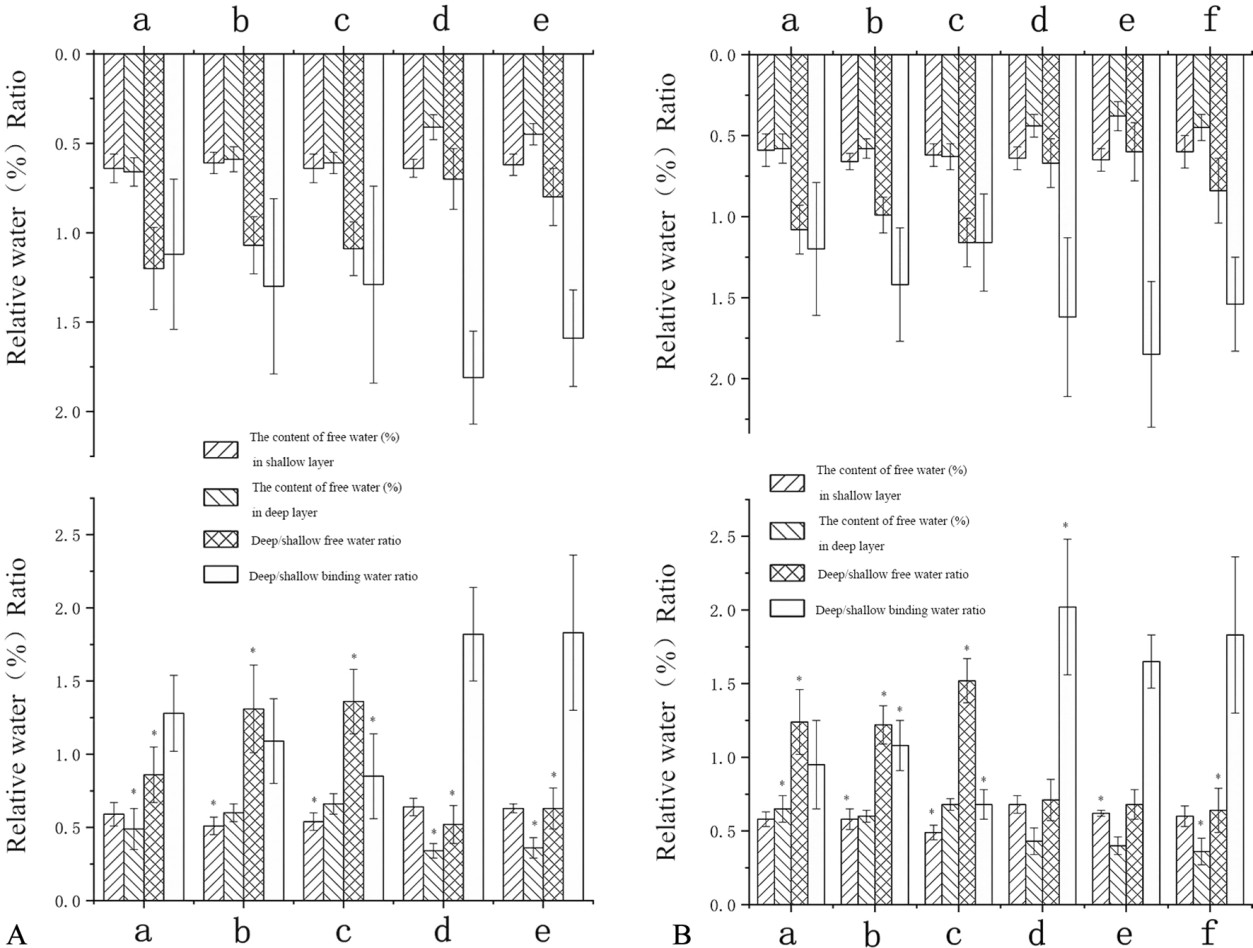
Histograms display statistically significant knee cartilages between patients without and those with genu recurvatum, in which one or more of the four indices had p < 0.05 (*). (**A**) Comparison of the healthy limbs in patients without genu recurvatum (top row) and those with genu recurvatum (bottom row). a, The femur cartilage at the anterior horn of the medial meniscus; b, the femur cartilage at the posterior horn of the medial meniscus; c, the femur cartilage at the posterior horn of the lateral meniscus; d, the tibia cartilage at the anterior horn of the lateral meniscus; e, the thickest load-bearing cartilage of the lateral tibial condyle. (**B**) Comparison of the hemiplegic limbs in patients without genu recurvatum (top row) and those with genu recurvatum (bottom row). a, The femur cartilage at the posterior horn of the medial meniscus; b, the femur cartilage at the anterior horn of the lateral meniscus; c, the femur cartilage at the posterior horn of the lateral meniscus; d, the tibia cartilage at the posterior horn of the medial meniscus; e, the tibia cartilage at the anterior horn of the lateral meniscus; f, the thickest load-bearing cartilage of the lateral tibial condyle.

In the healthy limbs, the deep free water content of the tibia cartilage at the anterior horn of the lateral meniscus, the femur cartilage at the anterior horn of the medial meniscus and the load-bearing region of the lateral condyle of the tibia cartilage in patients with genu recurvatum were higher than those in patients with no genu recurvatum. In patients with genu recurvatum, the free water in the shallow layer of the femur cartilage at the posterior horn of the medial meniscus and the femur cartilage at the posterior horn of the lateral meniscus were higher than in patients with no genu recurvatum. The deep binding water of the femur cartilage at the posterior horn of the lateral meniscus was higher than that in patients with no genu recurvatum (Fig. 2).

### Comparison between patients with mild and severe genu recurvatum

In the affected limbs, the deep free water content of the femur cartilages at the anterior and posterior horns of the medial meniscus and the load-bearing area of the lateral condyle of the femur in the patients with severe genu recurvatum were greater than those in patients with mild genu recurvatum. The deep binding water of the femur cartilages at the anterior horn of the medial meniscus and the load-bearing areas of the medial femoral condyle and medial tibial condyle were lower than those of patients with mild genu recurvatum. The free water content in the shallow layer of the tibia cartilage at the anterior horn of the lateral meniscus was less than that in the patients with mild genu recurvatum.

In the healthy limbs, the free water content of the femur cartilage shallow layer at the posterior horn of the medial meniscus in the patients with severe genu recurvatum was higher than that in patients with mild genu recurvatum, and the deep free water content of the femur cartilages at the anterior horn of the lateral meniscus and the load-bearing area of the medial condyle of the femur were higher than those in patients with mild genu recurvatum.

**Figure 3:**
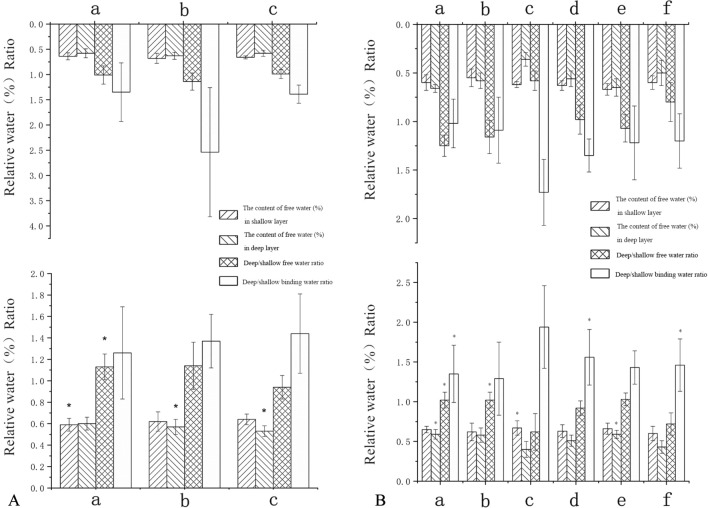
Histograms display statistically significant changes in knee cartilages between patients with mild genu recurvatum and those with severe genu recurvatum, in which one or more of the four indices had p < 0.05 (*). (**A**) Comparison of the healthy limbs of patients with mild genu recurvatum (top row) and those with severe genu recurvatum (bottom row). a, The femur cartilage at the posterior horn of the medial meniscus; b, the femur cartilage at the anterior horn of the lateral meniscus; c, the thickest load-bearing cartilage of the medial femoral condyle. (**B**) Comparison of the hemiplegic limbs in patients with mild genu recurvatum (top row) and those with severe genu recurvatum (bottom row). a, The femur cartilage at the anterior horn of the medial meniscus; b, the femur cartilage at the posterior horn of the medial meniscus; c, the tibia cartilage at the anterior horn of the lateral meniscus; d, the thickest load-bearing cartilage of the medial femoral condyle; e, the thickest load-bearing cartilage of the lateral femoral condyle; f, the thickest load-bearing cartilage of the medial tibia condyle.

The changes in these indices were statistically significant at *P* < 0.05 (Fig. 3).

## Discussion

The preliminary results showed that the knee cartilage of stroke patients had significant changes compared with the normal group, and the changes were more significant with increased genu recurvatum degree; both the healthy limb and the affected limb showed cartilage changes.

**Figure 4:**
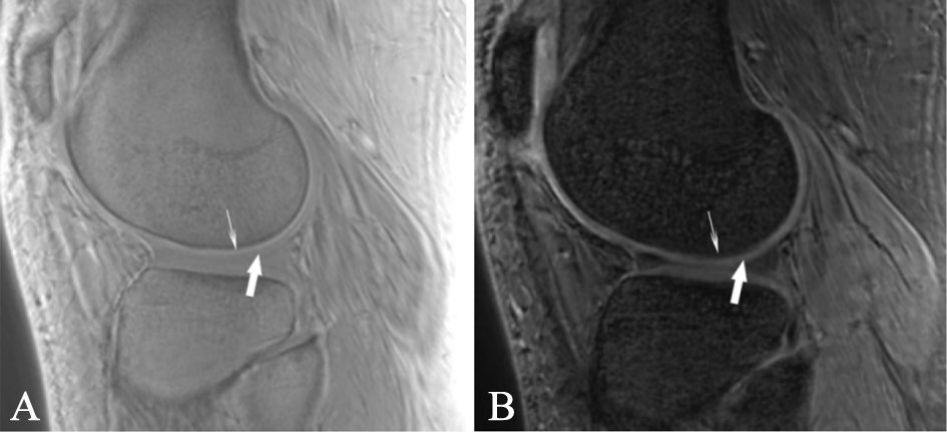
The appearance of cartilage on zero TE double-echo MR imaging. (**A**) On TE = 0 ms images, the calcified layer of the cartilage is a high-signal line (thin arrow) above the subchondral bone, while the other layers involved, including the superficial layer, the intermediate layer and the deep layer, are of intermediate signal intensity (thick arrow). (**B**) On TE = 4.6 ms images, the superficial layer, the intermediate layer and the deep layer of cartilage show high signal intensity (thick arrow), while the calcified layer of the cartilage and the subchondral bone are hypointense (thin arrow).

Articular cartilage is divided into four layers, starting from the articular surface, the superficial layer, the intermediate layer, the deep layer and the calcified layer, below which is the subchondral bone. Conventional magnetic resonance imaging cannot distinguish the cartilage calcification layer from subchondral bone^22,23^ and cannot accurately measure cartilage changes. As subchondral bone is the same as the bone cortex, the T1 relaxation time is short, and the proton density is low^24^, but the cartilage calcification layer is different; although it has the same short T1 relaxation time as subchondral bone, the proton density is high^25^. Although the proton density is very high, the T1 relaxation time of the upper deep cartilage is very long. Therefore, the calcification layer on UTE appears as a high signal line above the subchondral bone^26^ (Fig. 4). The high signal line is used to separate the cartilage layer from the subchondral layer, which avoids the influence of subchondral bone on the cartilage signal intensity and improves the accuracy of measurement.

Some MR techniques have been designed to evaluate the substance and its concentration^27–29^. For articular cartilage, there are three kinds of water molecules that can be investigated by the zero TE and UTE techniques. Eighty percent of the water is free water, with a long T2 value between 130 and 145 ms; 3% is bound to proteoglycan, with a T2 value between 8 and 12 ms; and 12% of the water is bound to collagen, with a T2 value between 3 and 18 ms. Multicomponent T2 analysis of articular cartilage^30^ yields similar results, with 6% bound to collagen, 14% bound to proteoglycan, and 80% free. The long T2* components obtained by UTE two-component analysis correspond to free water, while short T2* components indicate proteoglycan-bound water and collagen-bound water^26^. Since PD images can only be acquired for long TE tissues, the obtained signal intensity approximately corresponds to free water. Therefore, the changes in free water and bound water, such as those in extracellular matrix water, proteoglycan and fibrinogen at different stages of osteoarthropathy, could be obtained by comparison between the two. The water content in the surface layer of normal cartilage was abundant and decreased to the deep layer of cartilage, while the degree of aggregation of proteoglycans was the opposite^31^. The results showed that the content of binding water in the deep layer was higher than that of the shallow layer in all parts of the cartilage, and the free water content of the shallow layer in most parts of the cartilage was higher than that of the deep layer. The femoral cartilage at the anterior horn and posterior horn of the lateral meniscus or medial meniscus were the only locations in which the free water content of the deep layer was higher than that of the shallow layer. This might be related to stress, but the specific reasons need to be further studied.

From the onset of osteoarthritis, the proteoglycan and collagen fibrous network continues to undergo degenerative changes. During the development of osteoarthritis, the three kinds of extracellular matrix change correspondingly. The water content increases in the early stage of the disease and decreases in the middle and late stages. Proteoglycan rapidly decreases from the onset of osteoarthritis to later stages of the disease. Highly ordered collagen fibres become disordered in the early stage and seriously broken in the late stage^32–35^.

The affected and healthy limbs of hemiplegia patients were compared with the corresponding limbs of healthy volunteers. Both the affected and healthy limbs showed statistically significant differences in different parts of the cartilage, which basically showed a decrease in shallow free water content with or without a decrease in binding water or an increase or decrease in deep free water content and a decrease in binding water. These changes in the early and middle stages were consistent with the degeneration of articular cartilage. The different locations might be related to the changes in the mechanics of the affected and healthy limbs and the different mechanics of the cartilage, which need to be further explained in conjunction with mechanical analyses. Many studies have found significant asymmetry between hemiplegic limbs and nonhemiplegic limbs in stroke patients. The hemiplegic limbs have a slow gait rate, and their dysfunction leads to short standing durations, prolonged swinging durations, and reduced ground responses^36–38^, leading to insufficient power and poor progress. To adapt to this compensatory dysfunction, patients need to rely on the nonhemiplegic limb to maintain balance and push forwards, resulting in biomechanical changes of the nonhemiplegic limb, presenting with an erratic gait^39^. Patients prefer using the nonhemiplegic side limb to bear weight, resulting in asymmetry and interruption of the hemiplegic gait^40^. Therefore, healthy limb degenerative changes occur during the compensatory process, which was also confirmed by our experimental results. There were significant differences in some parts of the cartilage in the healthy limbs between the patients with hemiplegia and the healthy volunteers, which indicated that the so-called “healthy limbs” of the patients had also changed to a certain extent and could not be called truly healthy limbs. Therefore, in the process of making rehabilitation treatment plans, the “healthy” limbs should be considered together with the affected limbs.

In stroke patients, the femur cartilages at the anterior and posterior horn of the lateral meniscus and the tibia cartilages at the anterior and posterior horn of the medial meniscus in the knee of the healthy limb were different from those in the affected limb, indicating that the mechanical mechanics and walking modes of the healthy limb in patients were different to compensate for the function of the affected limb, leading to different degrees of damage.

Compared with that in patients with no genu recurvatum, the cartilage in both affected and healthy limbs of patients with genu recurvatum was significantly altered, showing increased shallow and/or deep free water, but the deep free water in the femur cartilage at the posterior horn of the medial meniscus of the affected limb was lower. Most of the changes in the cartilage involved decreased deep binding water content in patients with no genu recurvatum but only in the tibia cartilage at the posterior horn of the medial meniscus of the affected limb in the patients with genu recurvatum. Compared with that in patients with mild genu recurvatum, the deep free water content of the cartilage in the patients with severe genu recurvatum was increased, the binding water was decreased, and the free water in the shallow layer was decreased. Additionally, in patients with severe genu recurvatum, the deep free water content of the femur cartilage at the posterior horn of the medial meniscus was lower than that of the patients with mild genu recurvatum. Therefore, we can observe a trend: free water in the shallow and deep layers of the cartilage first increased and then decreased in the patients with no genu recurvatum, the patients with mild genu recurvatum and the patients with severe genu recurvatum, while the deep cartilage binding water continued to decrease in the patients with mild genu recurvatum and the patients with severe genu recurvatum. This trend was very similar to the trends in the degree of osteoarthropathy progression. At the early stage of cartilage injury, matrix metabolism is very active, matrix synthesis increases, and proteoglycan content is increased. Afterwards, the collagen grid is damaged, proteoglycan is lost, and the free water content is increased^41^. Hence, the knee joint changes caused by the degree of genu recurvatum are closely related to osteoarthropathy. If further study is combined with the onset time and walking time, it could be possible to further reveal the course and degree of knee joint osteoarthropathy caused by the degree of genu recurvatum.

This study had the following shortcomings: (1) the sample size was small, and large sample tests are needed for further verification; (2) signal strength conversion was adopted instead of the real data on free water and combined water, and the next step could involve verification by UTE T2* mapping; (3) sometimes the cartilage was thin, inevitably yielding some errors when measuring the deep and shallow layers; (4) there were no data on any mechanical changes in the knee joint with genu recurvatum, and these changes could not be perfectly explained.

## Subjects and methods

### Patient population

This study was approved by the Ethics Committee of the Beijing Rehabilitation Hospital (Protocol Number: 2020bkkyLW008). All methods were carried out in accordance with the relevant guidelines and regulations. Patients were hospitalized in the Neurological Rehabilitation Center of the Beijing Rehabilitation Hospital from January 2019 to October 2020, and healthy volunteers included staff members and students. All the patients and healthy volunteers in the study provided written informed consent.

Inclusion criteria: (1) initial onset of stroke, course of disease lasting more than 6 months, with hemiplegia on one side of the limb; (2) no previous history of knee injury; (3) ability to walk independently and safely at least 100 m and having walked for at least 3 months before inclusion, with a FAC grade III or above; (4) Brunnstrom stage III and above of the hemiplegic lower extremity; and (5) no obvious abnormal signals in the knee cartilages and meniscus on conventional T2W MRI and PD imaging.

Exclusion criteria: (1) obvious cognitive impairment (simple mental state checklist < 24 points), audio-visual comprehension disorder, or inability to cooperate; (2) neurological diseases that affect walking ability, such as involuntary movement, Parkinson's disease, tremor, etc.; (3) severe heart, lung, liver and renal insufficiency; (4) routine MRI findings of both knees indicating cartilage and meniscus injury; and (5) Claustrophobia and inability to complete MRI examination.

The loss of normal cartilage volume begins around the age of 40 years^42^, hence, healthy patients aged approximately 40 years were selected as the control group instead of age-matched healthy patients. Inclusion criteria for healthy volunteers: (1) age between 35 and 50 years old; (2) no history of knee osteoarthropathy; (3) no history of knee injury; (4) routine MRI showing no abnormal changes in the cartilage and meniscus.

### Imaging technique

All subjects were scanned with a GE Pioneer 3.0 T MRI scanner using a dedicated 16-channel knee surface coil and 3D dual-echo UTE sequence for MRI examination of both subjects' knees. The scanning parameters were as follows: TR/TE1/TE2, 12/0/4.6 ms; Matrix, 400 × 400; FOV 180 mm; Slice thickness 2.0 mm; Voxel volume 0.45 × 0.45 × 2.0 mm^3^; Flip Angle 8°; Half bandwidth 125 kHz; NEX 1. The positioning line was perpendicular to the posterior edge of the femoral internal and external condyles, and 20 layers of sagittal images were collected continuously. The scanning time of each knee joint was 8 m and 22 s.

### Imaging processing and data measurement

**Figure 5:**
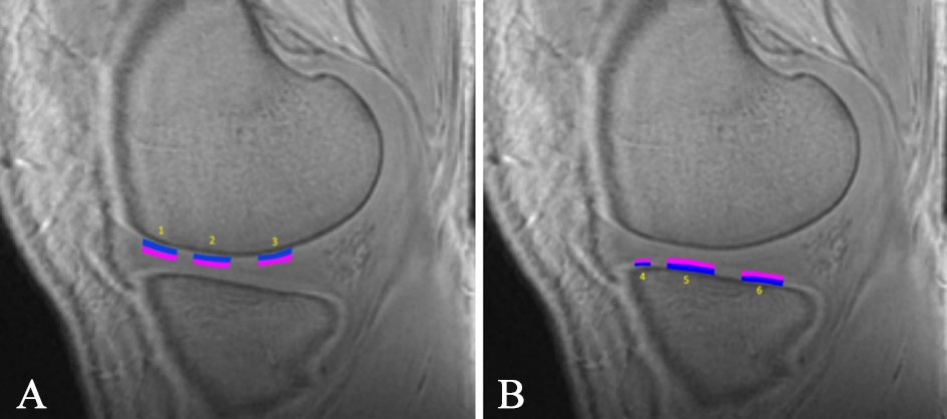
Cartilage position measured in this study. Zero TE MR imaging demonstrates the cartilage at the femur (**A**) and tibia (**B**) in the knee joint. The marked targets were measured. 1, The femur cartilage at the anterior horn of the medial (lateral) meniscus. 2, The cartilage at the load-bearing region of the medial (lateral) condyle of the femur. 3, The femur cartilage at the posterior horn of the medial (lateral) meniscus. 4, The tibia cartilage at the anterior horn of the medial (lateral) meniscus. 5, The cartilage at the load-bearing region of the medial (lateral) condyle of the tibia. 6, The tibia cartilage at the posterior horn of the medial (lateral) meniscus. The blue rectangle indicates the deep layer of cartilage, and the pink rectangle indicates the shallow layer of cartilage.

After acquisition, the data were transmitted to a GE AW4.6 workstation. The images were processed and measured by a radiologist with 16 years of experience in MR image processing. After selecting a patient or healthy volunteer, the TE = 0 images were first opened in the 3D synchroview for selection of the appropriate sagittal level in which the measured cartilage was the best displayed. The signal intensity of the cartilage at the thickest load-bearing point of the medial and lateral femoral condyles, the thickest load-bearing point of the medial and lateral tibial condyles, the femur cartilage at the anterior and posterior horn of the medial and lateral meniscus, and the tibia cartilage at the anterior and posterior horn of the medial and lateral meniscus were measured. The high signal intensity line of the calcified layer was used as the dividing line between the cartilage and subchondral bone. The thickness of the whole cartilage was measured first, and then the cartilage was divided into a shallow layer and a deep layer according to the Williams^43^ method (Fig. 5), with the centre of the cartilage thickness as the boundary. The shallow layer spanned the centre of the cartilage thickness to the articular surface, and the deep layer spanned the centre of the cartilage thickness to the high signal intensity line of the calcified layer. To avoid errors, an elliptical region of interest (ROI) was used. The ROI size was defined to cover the entire cartilage area to be measured. During the measurement of each position, the centre of the cartilage length was selected as the measurement area; the position was measured once before and after, and the value of the centre position was averaged as the final measurement value. The tibia cartilage at the anterior and posterior horns of the medial and lateral meniscus were measured differently. The cartilage here is very thin and difficult to measure. The length of half of the cartilage near the centre was taken for measurement, and the centre of the cartilage was used as the measurement point. Then, the TE = 4.6 ms images were opened and the above measurement process was repeated.

In the TE = 0 images, the signal intensity of the cartilage is mainly formed by the hydrogen protons of free water and bound water. In the TE = 4.6 ms images, the signal intensity of the cartilage is mainly formed by the hydrogen protons in free water. Therefore, the relative content of free water in the shallow and deep layers of the cartilage can be calculated according to the signal intensity, that is, the percentage of free water content = I (TE = 4.6 ms)/I (TE = 0 ms) × 100%, where I (TE = 4.6 ms) and I (TE = 0 ms) are the signal intensities in the TE = 4.6 ms and TE = 0 ms images, respectively, and then the ratio of free water in deep and shallow layers and the ratio of bound water could be calculated.

### Statistical analysis

Statistical analysis was performed using SPSS 17.0 statistical software. The cartilage at the 12 positions, including the thickest load-bearing cartilages of the medial and lateral femoral condyles, the thickest load-bearing cartilages of the medial and lateral tibial condyles, the femur cartilages at the anterior and posterior horn of the medial meniscus and lateral meniscus, and the tibia cartilages at the anterior and posterior horn of the medial meniscus and lateral meniscus, were compared between stroke patients with hemiplegia and healthy volunteers, between the healthy limb and paralyzed limb in stroke patients, and among stroke patients with no genu recurvatum and mild and severe genu recurvatum. The mean ± standard deviation is used to represent the data. The independent sample t test was used for all mean comparisons. When the data did not conform to a normal distribution or lacked homogeneity of variance, the Mann–Whitney test was used. *P* < 0.05 was considered statistically significant.

## Conclusion

Genu recurvatum in stroke patients with hemiplegia could cause changes in the moisture content of the knee cartilage. As the severity of the genu recurvatum increases, the changes in knee cartilage become more obvious. Both the affected limb and the healthy limb in stroke patients with hemiplegia exhibited corresponding changes. The so-called “healthy limb” may no longer be considered “healthy” and could not be used as a normal control; rather, it should be considered simultaneously with the affected limb in the development of a rehabilitation treatment plan.
